# Interrupted or continuous slowly absorbable sutures – Design of a multi-centre randomised trial to evaluate abdominal closure techniques INSECT-Trial [ISRCTN24023541]

**DOI:** 10.1186/1471-2482-5-3

**Published:** 2005-03-08

**Authors:** Hanns-Peter Knaebel, Moritz Koch, Stefan Sauerland, Markus K Diener, Markus W Büchler, Christoph M Seiler, the INSECT Study Group of the Study Centre of the German Surgical Society (SDGC)

**Affiliations:** 1 University of Heidelberg, Department of General, Visceral and Trauma Surgery, Im Neuenheimer Feld 110, D-69120 Heidelberg, Germany; 2 University of Cologne, Biochemical & Experimental Department, Ostmerheimer Str. 200, D-51109 Cologne, Germany

## Abstract

**Background:**

The closure of the abdomen after median laparotomy is still a matter of debate among surgeons. Further well designed and performed randomised controlled trials determining the optimal method of abdominal fascial closure are needed.

**Design:**

This is a three armed, multi-centre, intra-operatively randomised, controlled, patient blinded trial. Over 20 surgical departments will enrol 600 patients who are planned for an elective primary abdominal operation. The objective of this study is to compare the frequency of abdominal incisional hernias between two continuous suture techniques with different, slowly absorbable monofilament materials and an interrupted suture using an absorbable braided suture material at one year postoperatively.

**Conclusion:**

This trial will answer the question whether the continuous abdominal wall closure with a slowly absorbable material with longitudinal elasticity is superior to the continuous suture with a material lacking elasticity and to interrupted sutures with braided thread.

## Background

Median laparotomy is the most common technique of abdominal incisions because it is simple, provides adequate exposure to all four quadrants, is rapid to open and usually bloodsparing [1]. A major problem after median laparotomy remains the adequate technique of abdominal fascia closure. In prospective studies the incidence of incisional hernias varies from 9% to 20% [2,3]. Wound infection, obesity and suture closure technique are addressed as major risk factors for the development of an incisonal hernia [4,5].

Whereas patient related factors such as age, gender, body mass index (BMI), underlying disease, co-morbidities, prior surgical procedures and life-style factors (e. g. smoking) cannot be controlled or standardised, the decisive chance to lower the incidence of incisional hernias is to optimise the surgical technique. Therefore, a great variety of suture materials and needles has been developed to provide an adequate closure of the fascia and thus the abdominal wall. Thousands of patients have been included in trials in order to answer the question which is the optimal method in abdominal fascia closure and today a number of reviews and a meta-analysis are available. However, the reliability of the existing evidence is compromised by the low number of relevant randomised controlled trials (RCT's) [6-8].

Therefore the discussion regarding the optimal technique of abdominal fascia closure continues and most surgeons practice according to their own experience rather than acting evidence-based. This attitude resulted in an unchanged frequency of incisional hernias over the last decades [5]. None of the prior studies comparing rapidly absorbable braided materials with interrupted sutures versus slowly absorbable monofilament suture materials in a continuous technique were able to determine a definite superiority for one technique. Possible reasons may be small numbers of patients in each group or short follow-up [9-12].

We therefore conclude that there is a lack of data from a truly well-designed long-term trial performed in the daily practice of surgery. This has led us to develop a large randomised controlled trial comparing different surgical techniques of abdominal closure after median laparotomy. INSECT is a multi-centre, intraoperatively randomised controlled trial comparing three different standardised surgical techniques with certain needle/suture combinations on the occurrence of incisional hernia in patients with elective primary midline laparotomy. Two groups will use running sutures with different longitudinal elasticity (one group PDS™ and the other MonoPlus™) combined with an atraumatic needle and one group interrupted sutures (Vicryl™) with a traumatic needle. A three-group parallel equivalence design was selected because due to the results of the latest published meta-analysis [11] the superiority for one closure method has not been definitely proven. The randomisation procedure will be done stratified for participating centers. The planned sample size is at least 600 patients with a follow up period of three years.

INSECT is the first large-scale trial that started after a detailed theoretical and practical training of the participating surgical centers in March 2004 in order to reduce surgical bias in the study. INSECT will provide internal valid data for an adequate surgical technique of abdominal closure. Although we are well aware that this study cannot answer all open questions, the results should help to further improve evidence based surgery.

## Design

### Trial organization

INSECT has been designed and carried out by the Study Centre of the German Surgical Society (SDGC). The SDGC is an independent research group of the German Surgical Society and the Medical School of the University of Heidelberg that has to design, conduct and analyse large randomised surgical trials in order to improve daily surgical practice. The role of the sponsor (BBD Aesculap) is limited to material supply and local first-level-support. The sponsor is not involved in the database management and has no access to the randomisation code.

### Coordination

The trial is coordinated by the SDGC, which is responsible for overall trial management, trial registration (International Standard Randomised Controlled Trial Number (ISRCTN 24023541), ), database management, quality assurance including monitoring, reporting and for the scientific program of all trial related meetings.

### Investigators

Patients will be recruited by over 20 surgical centres in Germany. All investigators are hospital-based surgeons with a focus on general surgery. In order to obtain a representative trial result hospitals of all levels of care and education (county/community, private and university centres) are participating in this trial.

### Adverse events committee

This committee consists of 3 surgeons and decides on the final diagnostic classification of critical clinical events. For all serious adverse events the documentation and relevant patient data are verified by co-ordinating personnel of each centre before submitting the data to the Adverse Events Committee for diagnostic classification.

Burst abdomen, pulmonary infection and wound infection are secondary endpoints, but are also defined as Adverse Events (definitions see table 1). Burst abdomen and postoperative pulmonary infection will even be always a Serious Adverse Event. The term *Adverse Event *covers any sign, symptom, syndrome or illness that appears or worsens in a patient during the period of observation in the clinical trial and that may impair the well-being of the patient. The term also covers laboratory findings or results of other diagnostic procedures that are considered to be clinically relevant. A *Serious Adverse Event *is any adverse event that occurs at any time during the period of observation, that results in death, is immediately life-threatening, requires or prolongs hospitalisation, results in persistent or significant disability or incapacity.

**Table 1:** Definition of early onset and late complications

**Complication**	**Definition**
**Burst abdomen**	Postoperatively missing continuity of the abdominal fascia in combination with a wound dehiscence with consecutive relapse operation.
**Wound infection**	Redness, wound dehiscence with secretion either of putrid or caliginous, smelly fluid or requiring antibiotic treatment or surgical intervention.
**Postoperative pulmonary complication**	Infection of the lung with either evidence of increased infection parameters (CRP > 2 mg/dl and/or leukocytes> 10 0000/ml) which are not caused by a different pathologic process or evidence of pulmonary infiltration in the chest x-ray, requiring antibiotic therapy.
**Incisional hernia**	Postoperative evidence of a fascia dehiscence after completed superficial wound healing with or without prolapse of abdominal organs, confirmed by abdominal ultrasound.

Analysis of safety related data is performed with respect to frequency of:

• Serious Adverse Events and Adverse Events stratified by body-system

• Adverse Events stratified by severity

• Adverse Events stratified by causality.

### Study material supply

Study materials for all centres will be acquired by the BBD Aesculap company. Each type of suture material derives from a single batch to eliminate material inconsistencies. All materials are delivered to the participating centres by local representatives of the sponsor who also guarantee local first-level-support.

### On-site monitoring

During recruitment of patients each centre is monitored on site according to good clinical practice (GCP) guidelines. The data monitoring for this trial will be performed by an independent study nurse who is not involved in the trial or in completion of the case report form (CRF). The surgical monitoring will be done by independent surgeons being not involved in conducting this trial.

### Ethics, Informed Consent and Safety

The final protocol was approved by the ethics committee of the University of Heidelberg, Medical School. Secondary approval is gathered from all local ethics committees responsible for the participating centres. Informed consent will be obtained from each patient in oral and written form before inclusion in the trial.

### Patient selection

INSECT focuses on hospitalised patients over 18 years of age who are planned for an elective primary abdominal operation and are eligible for a vertical abdominal incision in order to perform the planned surgical procedure. A detailed overview of all eligibility criteria is given in Table 2.

**Table 2:** Eligibility Criteria

**Inclusion criteria**	**Exclusion criteria**
• Age equal or greater than 18 years • Expected survival time more than 12 months • Patients undergoing primary and elective median laparotomy (patients with prior laparoscopy or abdominal operation via paramedian incision (e.g. appendectomy) may be included in the trial) • BMI < 35 • Expected length of incision > 15 cm • Patient must be able to give informed consent • Patient has given informed consent	• Peritonitis • Emergency surgery • Participation in another intervention-trial with interference of intervention and outcome of this study • Coagulopathy A group of disorders of the blood clotting (coagulation) system in which bleeding is prolonged and excessive with abnormal values in the blood laboratory. • Severe psychiatric or neurologic diseases • Lack of compliance • Drug- and/or alcohol-abuse according to local standards • Current immunosuppressive therapy (more than 40 mg of a corticoid per day or azathioprin) • Chemotherapy within 2 weeks before operation • Radiotherapy of the abdomen completed longer than 8 weeks before operation • Inability to follow the instructions given by the investigator or the telephone interviewer (insufficient command of language, dementia, lack of time) • Lack of informed consent

### Study objectives

The primary objective of this study is to compare the frequency of incisional hernias between three different abdominal fascia closure methods after one year postoperatively: two continuous slowly absorbable monofilament suture materials with and without longitudinal elasticity respectively (MonoPlus™ USP 1, 150 cm loop, with a HRT-48 needle, BBD Aesculap Tuttlingen, Germany and PDS II™ USP 1, 150 cm loop, Ethicon Norderstedt, Germany) and a interrupted suture with an absorbable braided suture material (Vicryl™ USP 2, 6 × 45 cm, non-needled plus a traumatic needle, Ethicon Norderstedt, Germany).

Secondary objectives are the frequencies of early and late onset complications such as burst abdomen, postoperative pulmonary complications, wound infections and incisional hernias after three years postoperatively. Additionally a set of surgical and non-surgical parameters related to the operation will be analysed as secondary objectives such as the frequencies of various complications, the lung function and the postoperative length of hospital stay. A qualitative analysis is included in the study to assess the relevance of the primary endpoint from the patient's and the surgeon's perspective. The following aspects are ranked in a descending order from 1 (= most important) to 9 (= least important): postoperative complication, intraoperative complication, length of hospital stay, onset of enteral nutrition, death, postoperative pain, postoperative fatigue, convalescence of the complete physical maximum resilience and cosmetic result [13]. The ranking by the surgeon is done once for each surgeon before the operation. Patients are completing the ranking twice: first at inclusion and second at their discharge, to investigate if the patient's initial perspective changes within the hospital stay. Those various outcome parameters will be evaluated as part of an additional scientific project to build up a basis for further relevant questions in abdominal wall closure.

### Randomisation and surgical technique

A block-randomisation-list is generated via computer system (SAS Version 8.2, SAS Institute Inc., Cary, USA) and stratified for the individual centre. Each centre will contribute 30 patients (10 to each group). The sealed randomisation list is stored in the investigator file. Patients are randomised using sealed opaque envelopes in the operation theatre after the surgical procedure has been started and before the abdominal wall will be closed.

**Figure 1:**
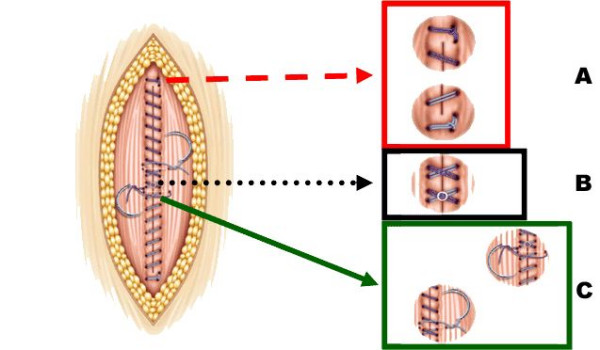
**Principles of continuous abdominal wall closure. **A Anchorage of the suture cranially/caudally outside the incision B Intersection of the loops in the middle of the incision C Knotting of each loop *Images reprinted with courtesy of Mrs. B. Wiehn, BBD Aesculap, Tuttlingen Germany*

Major challenge in this surgical trial compared to pharmaceutical trials is the standardisation of the surgical technique. All patients undergo a skin incision using electric cautery. Upon completion of the surgical procedure, closure of the abdominal wall is performed in all three groups in a standardised manner: four sharp Mikulicz-Clamps are placed at the corners of the incision and in the middle of the edges of the abdominal fascia and then the closure technique will be performed according to randomisation. Three groups are available: a continuous, all-layer closure technique with either two monofilament loops or an interrupted technique using a braided material (material as described above). In all three groups suturing is initiated at both ends of the incision towards the middle, whereas the continuous suture line is overlapping at the centre for at least 2 cm to secure each other (for details see figure 1). Neither a subcutaneous closure nor a subcutaneous drainage is to be inserted. Skin closure is done with skin clips. Measurement of the length of scar in centimeters (cm) is performed. A detailed description of the required surgical technique is given in the INSECT-study protocol enhanced by images and sketches as well as video material used during the investigator meeting and provided to all centres and investigators.

In an investigator meeting before trial initiation (March 5^th ^– 6^th^, 2004, AESCULAPIUM, Tuttlingen, Germany) all participating centres have been trained in the required techniques using abdominal wall models of mini-pigs. An evaluation of the investigator meeting was performed by all participants and will be published shortly. Investigators being unable to attend were trained on-site using the same training materials as in the investigator meeting. Training materials were supplemented by videos demonstrating the abdominal wall closure techniques (both continuous and interrupted) in the in vivo situation and in the animal model giving all sub-investigators good insight into the required techniques. Furthermore, a trial manager of the SDGC is available and responsible for all trial related issues and questions.

### Blinding

The patient is blinded for the technique of abdominal wall closure as the randomisation is performed intra-operatively. The patient will remain blinded until the assessment of the primary end-point at one year post-operatively. If feasible within the infrastructure of the participating centre, observers should be independent and not involved in completing the CRF and would thus also be blinded for the used technique.

### Follow up

Patients are observed for 30 days postoperatively for early onset complications defined as secondary endpoints such re-admittance for burst abdomen. One year after the operation the primary end-point will be assessed by documenting the incidence of incisional hernia with a physical examination and ultrasound of the abdominal wall. Follow up is completed 3 years after the primary operation to register any long-term complications of the used method for abdominal wall closure and to document any late-occurring hernias (see table 3 for detailed follow up).

**Table 3:** Flow Chart INSECT-Trial

**Visit**	**1 **(=Screening)	**2 **(OP)	**3 **(day 2 post OP)	**4 **(day of discharge)	**5 **(6 months +/- 1 month post OP)	**6 **(12 months +/- 1 month post OP)	**7 **(three years after operation)	**Extra-visit **(secondary endpoint, AE or SAE)
Past medical history	X							
Informed consent	X							
Physical examination including the personal data	X				X	X		
Basic study-related examination I (for each secondary endpoint, AE, SAE)	X (ranking of the patient before operation)	X (ranking of the surgeon)	X	X (ranking of the patient after operation)	X	X (ranking of the patient after operation)		X
Basic study-related examination II							X	
Ultrasound of the abdominal wall					X	X		X
Lung function test	X		X					
Medication	X				X	X		X

Past medical history: past medical history, past surgical history, indication for operation, diabetes, renal insufficiency, smoking, lung disease

Personal data: gender, date of birth, height in cm, weight in kg

Basic study related examination I: physical examination for evaluation of all secondary endpoints including adverse events/serious adverse events where appropriate

Basic study related examination II: telephone visit of patient and/or practitioner and physical re-examination if necessary in case of unclear abdominal wall status

Physical examination: vital signs (blood pressure systolic/diastolic in mmHg, heartrate in /min), prior abdominal incisions, rectus diastasis

Ultrasound: standard abdominal wall investigation (if hernia present: length and width in cm)

Lung function test: FEV, vital capacity %

### Data management and quality assurance

Investigators enter data directly in paper-based case report form (CRF). These are arranged for each visit time-point and contain instructions and relevant definitions. All treatments are recorded in treatment logs. Standard adverse events forms are used to document (serious) adverse events and relevant clinical procedures that have been carried out. After verification of the data entered according to "Good Clinical Practice" (GCP), one copy of each completed CRF is sent by mail to the SDGC. A concurrent database is maintained there. All incoming CRF are scanned to be electronically archived. Data are entered in a specially developed relational data base management system. The data entry module contains on-line range and logical checks. For data that are found missing, illegible or inconsistent, data clarification forms are generated which are sent to the on-site monitor for resolution.

Certain events must be reported immediately by the investigator by fax on preprinted forms directly to the coordinating centre. Examples are: informed consent and randomisation form, serious adverse events and premature withdrawal form the trial. The reporting of serious adverse events complies with national regulatory requirements.

### Statistical considerations and sample size estimation

Statistical methods are used to assess the quality of the data, homogeneity of treatment groups, endpoints and safety of the three different techniques.

The analysis is performed on the basis of an intention to treat (ITT) population and with respect to ITT principles. A patient belongs to the ITT population after the randomisation. The primary endpoint will also be analysed on the basis of a "per protocol" population.

To enable multiple comparisons in this three-armed study the closed testing procedure will be used[14]. All three treatment groups are considered separately without assuming any pre-specified monotonic trend among groups. All testing is done two-sided. The three elementary hypotheses address pairwise comparisons of the incisional hernia rates R_1_, R_2_, and R_3_. These hypotheses H_12_: R_1 _= R_2_, H_13_: R_1 _= R_3_, and H_23_: R_2 _= R_3_, are all contained in the global null hypothesis H_123_: R_1 _= R_2 _= R_3_. Sample size estimation, however, will be based on the elementary hypotheses, thus leading to sufficient power in the global test. Bauer had recommended that "sample sizes should be chosen large enough to give a high chance of jumping over the initial hurdle." [15].

The calculation of sample size is based on literature data, as summarised most recently by van 't Riet et al [11]. With interrupted suturing with an absorbable material, such as Vicryl^®^, incisional hernias were seen in about 13% of patients. This number is primarily based on the four-armed trial by Wissing et al. [16]. With the use of slowly absorbable materials, such as PDS^®^, MonoPlus^® ^or Maxon^®^, lower herniation rates were found, but this holds true only for continuous suturing. So far, only one trial has compared fast and slowly absorbable materials for interrupted sutures [17].

The comparison of interrupted rapidly absorbable versus continuous slowly absorbable sutures has been the aim of four previous studies. The results of these studies showed a non-significant tendency towards a lower rate of incisional hernia. Taking this data and the improved suture properties into consideration, a hernia incidence of 4% could be reasonably expected in one or both of the non-Vicryl^® ^groups over the first postoperative year. Smaller differences are also unlikely to be of clinical relevance.

**Figure 2:**
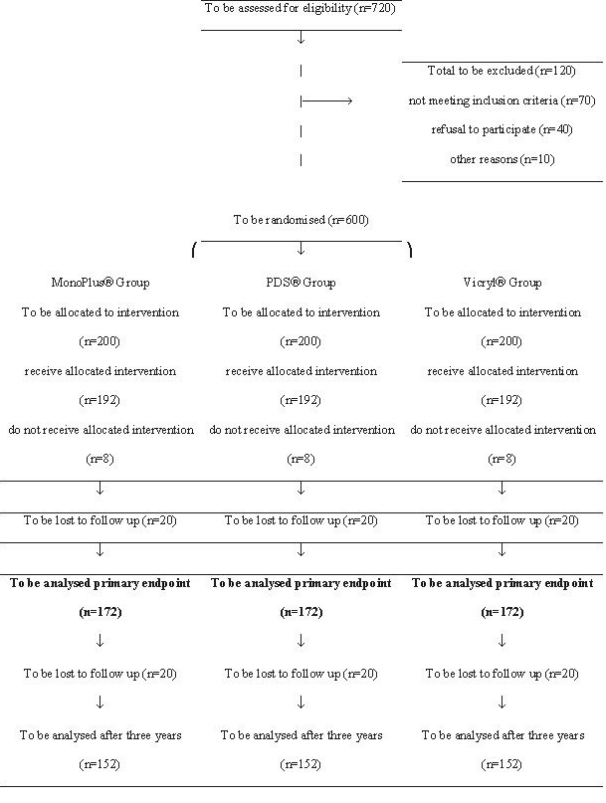
INSECT-Trial according to CONSORT (Moher et al. Lancet 2001)

Assuming annual hernia incidences of 13% and 4% in at least two of the groups, a raw sample size of 172 patients per group can be calculated with a two-sided alpha of 0.05 and a beta of 0.20 (employing Fisher's exact test). We used the PS power and sample size program of Dupont and Plummer (Version 1.0.17) for sample size estimation (freely available at: ). To account for an estimated 10% loss to follow-up and 2% surgical non-compliance with treatment allocation, sample size should be increased to 200 patients per group. In summary, the trial should recruit a total of 600 patients, with equal randomisation into the three groups. Thus a total of 720 patients have to be screened according to the CONSORT statement (Figure 2) [18].

In the primary intention-to-treat analysis, testing will start with the global null hypothesis H_123_: R_1 _= R_2 _= R_3_. Only if this global test is significant at the 0.05 level, the three elementary hypotheses H_12_: R_1 _= R_2_, H_13_: R_1 _= R_3_, and H_23_: R_2 _= R_3 _will be tested next. These pairwise comparisons (Vicryl^® ^versus PDS^®^, Vicryl^® ^versus MonoPlus^®^, and PDS^® ^versus MonoPlus^®^) use the same alpha level of 0.05, because closed test procedures in three-armed designs do not require alpha level adjustment. If, however, the first global hypothesis cannot be rejected at alpha level, the family of elementary hypotheses will not be tested at all, except for exploratory reasons.

To control for possible differences with regard to surgical procedures (colorectal vs. gastric or pancreatic operations), centre, and surgical expertise (board-certified surgeon vs. assistant surgeon), logistic regression will be used. We expect that the use of logistic regression will not essentially comprise power assumptions when compared to univariate testing. The inclusion of time-to-event data in the primary statistical analysis (e.g. by applying Kaplan-Meier-curves or Cox-regression) does not confer specific advantages and seems unwarranted, also because an incisional hernia causes similar consequences for the patient irregardless of whether the hernia occurred after 3, 6 or 9 months.

### Secondary endpoints, demographic and other variables

The analysis of the secondary endpoints and demographic variables will be descriptive.

The description of continuous variables includes at least: numbers of observations, mean, standard deviation, median, minimum and maximum. The description of categorical variables (ordinal or nominal) includes at least the number and percentage of patients belonging to the relevant categories in the trial population as well as in each treatment group.

The description of the ranking of parameters of interest (patient's view before, after operation (day of discharge and 12 months later) and surgeon's view) includes the mean and median for each category in the trial population. There will be a listing for all measurements taken for the patients. In order to improve the presentation of the observed data, graphical methods will be applied.

### Closing of the clinical database and follow up database

The clinical database including all information until 12 months after the operation will be closed six months after the last visit, complete documentation of all cases and resolution of all queries. At this time the primary endpoint of the trial will be ascertained. The information of the final follow-up three years after the operation will be added later. This database will be closed three months after the last telephone three years after the last operation.

### Current status and planning

The initial idea and hypothesis for the study was developed in March 2003. After a systematic review of the literature according to Cochrane standards has been performed the study protocol was completed in October 2003. The study protocol was approved by the local ethics committee of the University of Heidelberg in December 2003. Preparation of all study and instructional materials (including video clips) was completed in February 2004 and the first investigator meeting was held in Tuttlingen, Germany on March 5^th ^and 6^th^, 2004. In June 2004, following completion of contracts and approval of local ethics committees, the first centres were initiated and the first patient was recruited in July 2004. Currently 23 centres are in an active status recruiting the required 30 patients per centre. Further 6 centres are processed and will be activated within the next months. Assuming an enrolment of 5 patients per month and centre the end of recruitment is assumed to be in October 2005.

## Discussion

The strategy to publish study protocols in surgery and thus to enhance transparency in a clinical trial might be relatively new in surgery, but has been practised in other medical fields for several years [19,20]. This mentioned transparency increases reliability and validity of the results when a detailed description of the experiment is published prior to the conduction. The assumption how many patients should be screened to include the sufficient number of patients based on the sample size calculation needs to be transparent in order to evaluate whether results from this trial are transferable to daily practice. Therefore a flow chart according to the CONSORT statement should be included in study protocols (Figure 1). However, many important details of a randomised surgical trial are interesting to the reader and can not be published together with the final results. It also seems out of the question that medical journals that surgical trials should be internationally registered [21].

INSECT is designed to help answering the question which is the optimal method for abdominal wall closure. Although there are a substantial number of randomised studies and several meta-analyses examining different techniques of abdominal fascia closure the optimal and definite method for closing the abdomen has not yet been found [6,7,11]. Therefore, the technique and materials for abdominal wall closure are still determined by local material supply and surgical tradition. These preconditions and a detailed literature research gave us reason to design a trial comparing three standardised common techniques of abdominal fascia closure in abdominal surgery.

Several design features of INSECT were discussed before the trial started. A two armed or a three armed study design was debated before writing the protocol. As we wanted to compare the clinically most relevant and most evidence-based suture materials and suture techniques for closure of midline abdominal incisions the most recent and most relevant meta-analysis as a data-base for choosing the different groups in our trial was used [11]. Two different suture techniques and materials became clear to show the best results in abdominal fascia closure and revealed the lowest incidence for incisional hernias: slowly absorbable continuous (or running) sutures and absorbable interrupted sutures. Aside from those two groups of suture techniques (continuous versus interrupted sutures) we included a third group using the same technique as one of the other groups (continuous suture) but using a different suture material (monofilament suture with longitudinal elasticity) which is supplied by a different company. INSECT depicts the surgical reality and variety of abdominal wall closure as it compares not only different suture techniques but also several suture materials offered by different companies. Therefore, the results of INSECT will be relevant for the majority of surgeons as the materials and techniques used in this trial are widely accepted in daily use.

An important issue for a good trial is to define adequate and well designed end-points. Regarding the optimal method for abdominal closure a great variety of objectives has to be considered: wound infection, wound dehiscence, incisional hernia, suture sinus, wound pain, etc. The socio-economic and surgical most relevant objective is the incidence of incisional hernias [4]. Because 50–70% of incisional hernias will occur within one year after operation we selected the frequency of incisional hernias at one year postoperatively as the primary end-point of our study. All other relevant objectives such as wound infection, pain, pulmonary impairment, etc. were included as secondary endpoints into the trial design. Additionally a qualitative analysis for surgeons and patients was planned to evaluate the importance of different outcome variables between surgeons and patients.

A major challenge in this INSECT trial compared to other studies, e. g. pharmaceutical trials, is the standardisation of the surgical technique. Lack of standardisation in surgical trials is often an argument of opponents who state that the achieved trial results would not be transferable to surgical routine. Therefore all patients in the INSECT trial undergo a standardised skin incision and opening of the abdominal cavity as well as the closure of the abdominal wall is performed in all three groups in a standardised manner. To provide an overall highly standardised surgical procedure in every study site all participating centres have been trained in the required techniques using abdominal wall models of mini-pigs during an investigator meeting before trial initiation. For investigators who were unable to attend the meeting training materials and videos demonstrating the different abdominal wall closure techniques (both continuous and interrupted) used in the study were provided. For further quality assurance there will be a regular surgical monitoring during the trial. Additionally, randomisation is stratified for each individual centre and the trial results will be therefore comparable among the different participating centres.

## Conclusion

Over the last years, surgeons' attitude towards randomised surgical trials has changed. It becomes more and more evident that surgical procedures could also be tested in randomised studies. Surgical expertise is largely a personal conviction and an apprenticeship with surgical techniques passed from one surgical generation to the next [6,22]. Most surgeons rather use particular techniques because they are trained in than using the most evidence-based technique. The Study Centre of the German Surgical Society (SDGC) was founded 2003 in order to ensure that surgical techniques are more evidence based and not simply the result of a surgical dogma. This is the first multi-centre trial designed and conducted by the SDGC after a special Study Group was founded. We are aware of the difficulties and problems of performing large surgical multi-centre trials and, therefore, we decided to start with a trial examining a fundamental, "simple" and daily performed surgical technique. As the abdominal fascia closure is largely based on tradition rather than evidence, the results of the INSECT trial will help to create more evidence and to guide surgeons to a critical review of their surgical routine.

## Abbreviations

AWC Abdominal wall closure

CRF Case Report Form

GCP Good Clinical Practice

ICH International Conference on Harmonisation of Technical

Requirements for Registration of Pharmaceuticals for Human Use

IEC Independent Ethics Committee

ITT Intention-to-treat-analysis

PDS^® ^Polydioxanone (absorbable suture material)

RCT Randomised Controlled Trial

SDGC Study Centre of the German Surgical Society

## Competing interests

The author(s) declare that they have no competing interests.

## Authors' contributions

Hanns-Peter Knaebel and Moritz Koch designed and wrote the manuscript, Stefan Sauerland prepared bio-statistics of the trial and is in charge of the data management, Markus K Diener searched and assessed the literature, Markus W Büchler sponsors the trial and provides necessary infrastructure, Christoph M Seiler searched and assessed the literature and overlooked the completion of the manuscript.

## Pre-publication history

The pre-publication history for this paper can be accessed here:


